# The Diagnostic Value of circFBXW7, circABCB10, and circ0103552 Levels in Breast Cancer

**DOI:** 10.3390/cimb46120862

**Published:** 2024-12-20

**Authors:** Burak İlhan, Şenol Ender, Berkay Kılıç, Muhammed Üçüncü, Murat Serilmez, Ceren Tilgen Yasasever, Hilal Oğuz Soydinç, Sibel Kuras, Bekir Erdoğan, Hani Alsaadoni, Hasan Karanlık, Süleyman Bademler

**Affiliations:** 1 Department of Surgery, Istanbul Faculty of Medicine, Istanbul University, Istanbul 34093, Türkiye; burak.ilhan@istanbul.edu.tr (B.İ.); shenolender@hotmail.com (Ş.E.); 2 Department of Surgery, Oncology Institute, Istanbul University, Istanbul 34093, Türkiye; berkaykilic28@yahoo.com (B.K.); hasankaranlik@yahoo.com (H.K.); 3 Department of Anesthesia, Vocational School of Health Services, Istanbul Gelişim University, Istanbul 34310, Türkiye; muhammeducuncu@gmail.com; 4 Department of Basic Oncology, Institute of Oncology, Istanbul University, Istanbul 34093, Türkiye; murat.serilmez@istanbul.edu.tr (M.S.); ceren.yasasever@istanbul.edu.tr (C.T.Y.); hoguz@istanbul.edu.tr (H.O.S.); 5 Department of Medical Biochemistry, Hamidiye Faculty of Medicine, University of Health Sciences, Istanbul 34668, Türkiye; sibel.kuras@sbu.edu.tr; 6 Department of Physiology, Hamidiye Faculty of Medicine, University of Health Sciences, Istanbul 34668, Türkiye; bekir.erdogan@sbu.edu.tr; 7 Department of Medical Biology, International School of Medicine, University of Health Sciences, Istanbul 34668, Türkiye; hani.alsaadoni@sbu.edu.tr

**Keywords:** circFBXW7, circABCB10, circ0103552, circRNA, breast cancer

## Abstract

Despite advances in cancer treatment, breast cancer (BC) remains one of the most common cancers affecting women worldwide. This study aimed to determine serum circFBXW7, circABCB10, and circ0103552 levels and compare BC patients and healthy controls to investigate their roles in the molecular mechanism of BC and the significance of these circRNAs in BC diagnosis. The study group consisted of 92 patients with BC and 31 healthy controls. Total RNA was isolated from serum samples. Following total RNA, complementary DNA was synthesized from this material. Following complementary DNA analysis, the circRNA levels were analyzed by the qRT-PCR method. Expression levels were evaluated in ΔCt values. High ΔCt values of circFBXW7 and circ0103552 and low ΔCt values of circABCB10 were correlated with BC diagnosis (circFBXW7, *p* = 0.043, r = 0.183, circ0103552, *p* < 0.001, r = 0.321, circABCB10, *p* = 0.001, r = −0.291). According to Fold Change (FC) values, circFBXW7 (FC = 0.30) and circ0103552 (FC = 0.26) showed low expression in the patient group compared to the control group, while circABCB10 (FC = 11.09) showed high expression (*p* < 0.05, for all comparisons). We think that our study is one of the rare studies investigating the relationship between BC and serum circRNA levels. This study concludes that the significant downregulation of circFBXW7 and circ0103552 and the upregulation of circABCB10 are directly related to the diagnosis of BC and can be used for diagnosis, but further studies are needed to elucidate the molecular mechanism of the relationship between circRNAs and BC.

## 1. Introduction

Current screening programs and recent advances in imaging technologies have enabled physicians to detect breast cancer (BC) in the early stages, but many patients still present with advanced cancer, which is a critical cause of mortality. According to data from the Global Cancer Statistics 2022, lung malignancies are the most frequently diagnosed cancer globally, followed by female BC with 2.3 million new cases. BC accounts for 11.6% of all cancer cases and ranks 4th in mortality rates, with 6.9% among all cancers [1]. BC is considered a heterogeneous carcinoma with various molecular subtypes [2]. The fact that BC is such a heterogeneous malignancy makes it complicated to select diagnostic and therapeutic options and to predict disease progression and survival [3]. Consequently, understanding more effective molecular mechanisms and developing diagnostic biomarkers are becoming increasingly important for clinicians in BC diagnosis, treatment, and screening. In these circumstances, circular RNAs (circRNAs) have emerged as an intriguing new class of groundbreaking and non-invasive early diagnostic, prognostic biomarkers [4]. circRNAs are special subtypes of noncoding RNA (ncRNA), identified in the mid-1970s and characterized by a loop structure without free 3′ and 5′ ends [5,6]. circRNAs have regulatory and cell function task interactions and have many biological effects, including sponging microRNAs (miRNA), transcriptional regulation or splicing, and communication with RNA-binding proteins [7]. Although initially thought to be insignificant biomolecules derived from splicing errors, current studies have shown that like many other kinds of ncRNAs, circRNAs play critical roles in diseases such as neurological disorders, cardiovascular diseases, and diabetes, as well as tumorigenesis, metastasis, cancer recurrence, and multidrug resistance [4].

circRNAs have roles in promoting proliferation, migration, and apoptosis by various activities [8]. The variable expressions of circRNAs are known to be involved in almost all cancer types and have been implicated as oncogenes in carcinogenesis [9]. Similarly, circRNAs have promoting or inhibitory effects on carcinogenesis in BC. One of these circRNAs in BC, circ0006528, activates miR-7-5p-sponge to regulate Raf1 expression, which directs the MAPK/ERK signaling pathway and stimulates carcinogenesis [10]. Another study showed that elevated circACAP2 expression in BC tissues induces proliferation and motility of BC cells by miR-29a/b-3p-sponge and comminating COL5A1 [11]. In contrast, circRNA-000911 limits carcinogenic features of cells and allows apoptosis of BC cells by capturing miR-449a to inhibit nuclear factor-κB signaling and improve Notch1 gene expression [12].

circFBXW7 (hsa-circ0001451), one of the circRNAs that was the main element of our study, was discovered to be a circRNA encoding a tumor suppressor protein in glioma, containing a circular F-box and WD repeat domain. In addition, FBXW7 acts as a tumor suppressor by controlling proteasome-mediated degradation of many oncoproteins such as cyclin E, Mcl-1, Jun, Notch, and AURKA. In addition, the chromosomal location is in a genomic region deleted in more than 30% of all human cancers [13]. circFBXW7 is an antioncogenic product encoded by the Fbxw7 protein that acts as a tumor suppressor [14]. The negative correlation of circFBXW7 expression has been associated with increased tumor size and lymph node spread as an independent prognostic risk factor in BC and especially in triple-negative breast cancer (TNBC) [15].

The mTOR molecule in the mammalian target of rapamycin complex (mTOR) signaling pathway, one of the signaling pathways whose role in cancer is well known, functions primarily as a downstream signaling molecule of the AMP-activated protein kinase (AMPK) pathway, mitogen-activated protein kinase (MAPK) pathway, and phosphatidylinositol 3-kinase (PI3K)/protein kinase B (Akt) signaling pathways and induces cell growth, survival, proliferation, and migration. In addition, phosphatase and tensin homolog (PTEN), one of the downstream molecules of the PI3K/Akt signaling pathway, is a tumor-suppressor protein. Studies have revealed that circRNA FBXW7 not only inhibits the formation and progression of gliomas, but also inhibits the malignant progression of colorectal cancer and gastric cancer by inhibiting mTOR expression by activating PTEN [16].

circABCB10 (hsa-circ0008717) originates from exons 2 and 3 of the ABCB10 gene located on chromosome 1. It is located at chr1:229665945-229678118 with a length of 724 nucleotides in the ABCB10 gene symbol, hence the name circABCB10. The studies in the literature revealed that the knockdown of circABCB10 indicates suppressed proliferation and enhanced apoptosis in BC cells [17]. Overall, circABCB10 has the potential to function as an oncogene by driving tumor proliferation and migration in cancers such as glioma, non-small cell lung cancer, esophageal cancer, breast cancer, and renal cancer [18,19,20,21,22].

circ0103552 is a circRNA that maps to chr15:43294752-43314999 in the human genome. The spliced length consists of 920 nucleotides [23]. Yang L et al. reported that circ0103552 levels elevated in BC cells and tissues. According to this study, lower survival rates in BC patients are significantly related to its expression. It has also been shown that high expression of circ0103552 may result in unfavorable pathologic features and poor prognosis in BC patients [24].

Although studies in the literature report that these three circRNAs have individual roles in BC development and its progression, there are limited studies on their use for diagnosis. Therefore, it is of critical importance, as it is a breakthrough. In this study, we aimed to investigate the serum expression levels and characteristics of these circRNAs regarding the effectiveness of their diagnostic use.

## 2. Materials and Methods

Our study consisted of 123 individuals who visited our tertiary hospital for screening and treatment between January 2023 and June 2023. Of these, 92 people diagnosed with BC were identified as our patient group. The remaining 31 individuals who were not diagnosed with cancer and who visited for routine screening without any finding of suspicious breast lesions constituted the control group. Patients with metastatic BC, patients with inconsistent pathology reports, individuals with another previous malignancy, and individuals who had concerns about participating and refrained from giving consent to participate were excluded from this study. The BC subtype of the majority of patients was invasive ductal carcinoma. To measure the identified circRNA levels, patients with initially operable cancer provided blood samples preoperatively, while advanced-stage BC patients scheduling neoadjuvant treatment obtained blood samples before the treatment.

This prospective study was conducted with the ethics committee approval numbered 2022/356, obtained from Istanbul University Istanbul Medical Faculty Medical Research Ethics Committee. Written informed consent was obtained from all participants.

### 2.1. Measurement of circRNA Levels and Determination of circRNA Expression

Total RNA isolation was performed from serum samples collected for total RNA isolation using Qiazol (Qiagen, Cat No: 79306, Dusseldorf, Germany), a guanidium thiocyanate solution. The concentrations of total RNA samples isolated and stored at −80 °C until this stage were first equalized. Then, complementary DNA (cDNA) was synthesized using the Quantitect Reverse Transcription Kit (Qiagen, Catalog No: 205311, Dusseldorf, Germany). According to the equalized RNA concentration for each sample, 12 µL of a mixture of RNase-free water and total RNA was placed in 0.2 µL tubes, and 2 µL of 7× gDNA Wipeout Buffer was added and vortexed. This mixture was incubated at 42 °C for 2 min. Then 1 µL Quantiscript Reverse Transcriptase enzyme, 4 µL 5× Quantiscript RT Buffer, and 1 µL RT Primer Mix were added to this mixture and vortexed. Finally, this mixture was incubated in a C1000 Touch Thermal Cycler, (Bio-Rad, Hercules, CA, USA) at 42 °C for 15 min, then at 95 °C for 3 min to inactivate the reaction, and stored at −20 °C until the next experimental step. After cDNA synthesis, the expression levels of circABCB10, circFBXW7, and circ0103552 RNAs were analyzed by the qRT-PCR system, CFX96 Touch, (Bio-Rad, Hercules, CA, USA) using specific primers. The β-Actin gene was used as a housekeeping gene.

In the analysis of qRT-PCR data, relative expression was calculated by comparing the PCR signal of the target region in the patient group to the PCR signal in the control group. Changes in Ct values obtained from the device were analyzed using the ΔΔCT method. Normalization calculations of expression analysis of circRNA genes were performed with housekeeping ß-Actin using the following formula:

ΔCt = Ct (target gene) − Ct (reference gene), ΔΔCt = mean ΔCt (patient group) − mean ΔCt (control group). The change in the expression level of a studied gene compared to the control group is called a Fold Change (FC). The expression level alternations of a gene were calculated with the formula FC = 2^−ΔΔCt^ [25].

### 2.2. Statistical Analysis

The Statistical Package for the Social Sciences (SPSS) program, version 22.0 (IBM Corp., Chicago, IL, USA), was used in the statistical analyses. The normal distribution of continuous variables was examined by the Kolmogorov–Smirnov test. The Pearson correlation test examined relationships of non-parametric variables between the groups. The Mann–Whitney U and Student’s T-test assessed comparisons between patients and controls regarding circRNA levels. The ROC (receiver operator characteristics curve) analyses were used to investigate the diagnostic availability of the parameters. This study considered a *p*-value of less than 0.05 statistically significant.

## 3. Results

**Figure 1:**
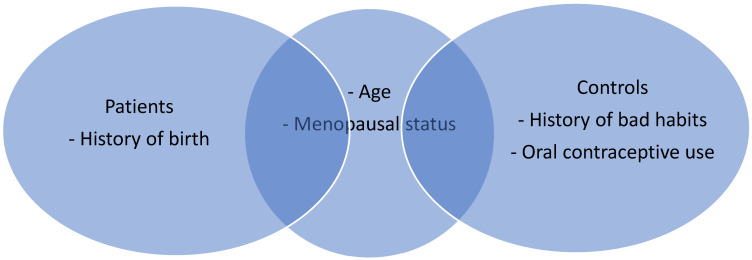
The distribution of patient-specific characteristics between patient and control groups.

**Figure 2:**
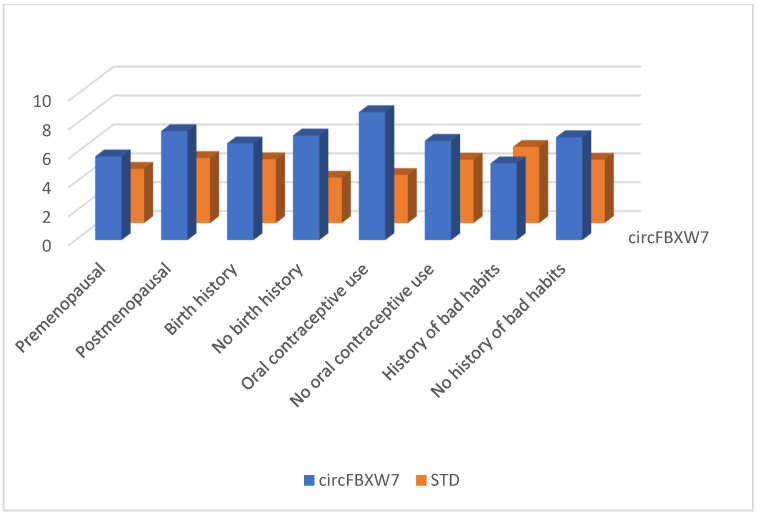
circFBXW7 levels regarding patient-specific characteristics.

**Figure 3:**
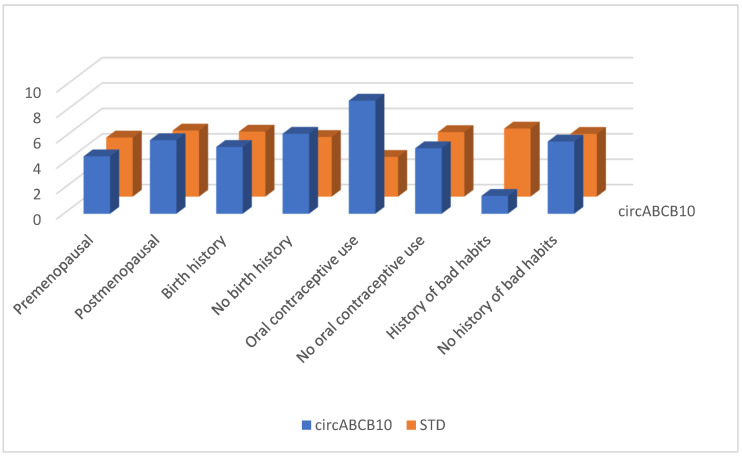
circABCB10 levels regarding patient-specific characteristics.

**Figure 4:**
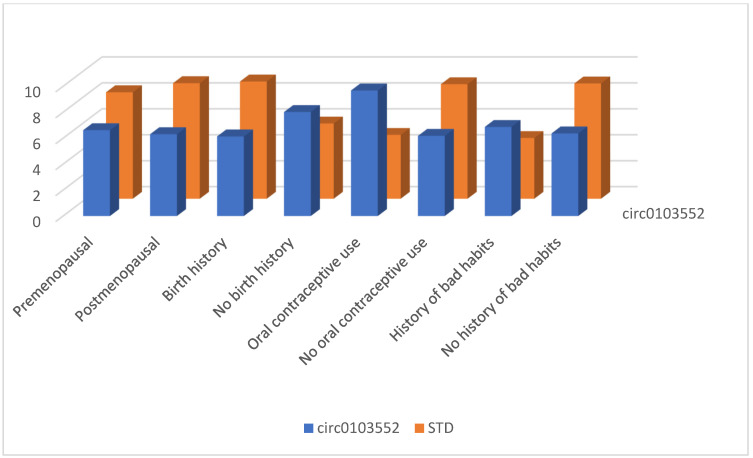
circ0103552 levels regarding patient-specific characteristics.

Between January and June 2023, 123 persons enrolled in this study. In our study group, 58 (47.1%) individuals aged <50 years and 65 (52.9%) individuals aged ≥50 years were included. Forty-six women (37.4%) were premenopausal, and 77 (62.6%) were postmenopausal. Ninety-five (77.2%) women had at least one birth history, while 28 (22.8%) did not. Nineteen (15.5%) patients had a history of oral contraceptive use, while 104 (84.5%) did not. A history of bad habits (smoking, alcohol, substance abuse) was present in 20 (16.3%) and absent in 103 (83.7%) persons. The distribution of patient-specific characteristics between patient and control groups is shown in Figure 1. There was no relationship between circRNA levels and these patient-specific characteristics such as varying age, menopause, fertility, oral contraceptive use, and history of bad habits (Figure 2, Figure 3 and Figure 4) (*p* > 0.05 for each comparison). Although oral contraceptive use and a history of bad habits were significant for circABCB10 in its figure, they were not interpreted as substantial due to the small number of these patients. However, high ΔCt values of circFBXW7, circ0103552, and low ΔCt values of circABCB10 were significantly correlated with BC diagnosis (circFBXW7, *p* = 0.043, r = 0.183, circ0103552, *p* < 0.001, r = 0.321, circABCB10, *p* = 0.001, r = −0.291).

In the patient group, there were 35 (38.0%) patients aged <50 years and 57 (62.0%) patients aged ≥50 years. The mean size of the tumor in the patient group was 36.53 mm (range 6 to 96 mm). According to the clinical T stage, there were 65 (70.7%) patients with T1-T2 and 27 (29.3%) with T3. Fifty-two (56.5%) patients were axilla negative, while 40 (43.5%) were positive. According to receptor profiles, 76 (82.6%) patients had luminal, 7 (7.6%) patients had HER2/neu, and 9 (9.7%) had triple-negative receptor status. There was no association between patient age, tumor features, and circRNA levels (Table 1) (*p* > 0.05 for each comparison).

**Figure 5:**
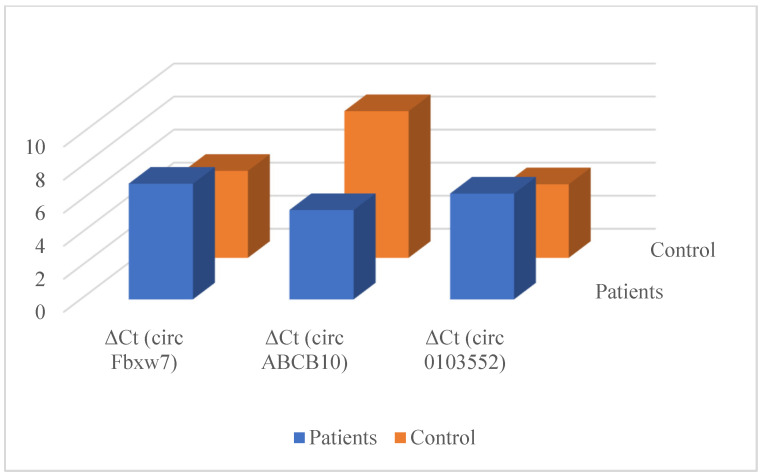
ΔCt values of circRNAs in patients with breast cancer and in healthy controls.

The mean circFBVW7 (7.00 vs. 5.26) and circ0103552 (6.41 vs. 4.45) levels were significantly higher and the circABCB10 (5.41 vs. 8.88) levels were lower in the patient group than in controls (*p* < 0.05 for each comparison). According to Fold Change (FC) values, circFBXW7 (FC = 0.30) and circ0103552 (FC = 0.26) showed low expression in the patient group compared to the control group, while circABCB10 (FC = 11.09) showed high expression (Table 2, Figure 5).

**Figure 6:**
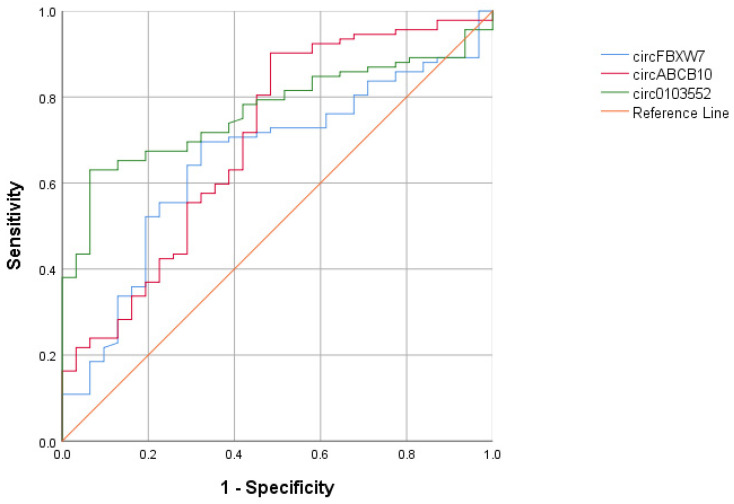
The receiver operating characteristic (ROC) analysis for serum circFBXW7, circABCB10, and circ0103552 levels.

circ0103552 had more effective positive and negative predictive values according to ROC analysis regarding its diagnostic significance. In this model, the sensitivity, specificity, and positive and negative predictive values of circ0103552 were 87.1%, 67.7%, 88.9%, and 54.7%, respectively (Table 3, Figure 6).

In the model where the cut-off values of circRNAs were taken as reference, we determined that the area under the curve (AUC) reached up to 90.8% when circRNAs were used in combination (CI: 80.3–97.8%) (Table 4).

## 4. Discussion

As in all cancers, early diagnosis remains critically important to reduce treatment-related mortality and prevent mortality in BC. Over time, many diagnostic biomarkers thought to be involved in cancer development have been investigated, and the circRNA axis has been prominently highlighted as a target for future perspectives. Generally, circRNA level studies in BC patients are limited in number as in other cancer types. Based on existing studies, the circRNA-miRNA-mRNA axis is closely associated with the progression of many types of cancer, including BC, in the community. According to the competitive endogenous RNA (ceRNA) theory, circRNAs may play the role of mRNA expression regulation by sponging miRNAs. circRNAs act as molecular sponges by absorbing miRNAs or serving as scaffolds for RNA-binding proteins, thereby modulating key biological processes involved in cancer initiation and progression [26]. Therefore, the circRNA-miRNA-mRNA axis is thought to be a common molecular mechanism in tumor pathogenesis and affects the pathogenesis of BC at the transcription level [27]. Their importance is increasing daily because they act as oncogenes or tumor-suppressive factors. This study is one of the rare studies aiming to investigate whether circFBXW7, circABCB10, and circ0103552 have a significant value in diagnosing BC.

circFBXW7 (hsa-circ0001451) was discovered to have a tumor suppressor effect encoding a novel protein in cerebral glioma. In addition, circFBXW7 is an antioncogenic product encoded by the Fbxw7 protein that acts as a tumor suppressor, and circFBXW7 is down-regulated as a result of mutation in hepatocellular carcinoma (HCC) and esophageal cancer, clarifying the role of Fbxw7 in the mechanism of HCC development [28,29]. Cao C et al. reported that circFBXW7 inhibits colorectal cancer formation and progression by activating PTEN and inhibiting mTOR (sirolimus) expression in colorectal carcinoma [30]. In two different studies, it was similarly shown that circFBXW7 inhibited proliferation, migration, and invasion in lung adenocarcinoma cells by showing a sponge effect on mir-492 and miR-942 [31,32]. Furthermore, some studies have revealed that circFBXW7 is down-regulated in T-cell Acute Lymphoblastic Leukemia (T-ALL), which promotes cell proliferation, invasion, and disease progression in T-ALL patients. Low expression of circFBXW7 increases MYC and intracellular NOTCH1 protein activation, which is associated with disease severity. These findings suggested that circFBXW7 has a tumor suppressor effect and may be helpful for the diagnosis and treatment of T-ALL and similar diseases [14]. Tayel et al. found that the expression levels of circFBXW7 were significantly reduced in acute myeloid leukemia (AML) compared to the control group. Additionally, other studies have reported that circFBXW7 functions as a tumor suppressor [33]. In a study conducted in colorectal cancer, circFBXW7 was shown to improve the resistance to chemotherapeutic agents in colorectal cancer by targeting and sponging miR-18b-5p and may be a promising strategy for patients [34]. Regarding the diagnosis and progression of BC, Ye et al. revealed that circFBXW7 acts as a miR-197-3p sponge and reduces TNBC progression and spread by upregulating FBXW7 expression. In addition, the Fbxw7-185aa protein encoded by circFBXW7 was found to decrease the proliferation and migratory capabilities of cancer cells by enhancing FBXW7 density and increasing c-Myc degradation. The negative correlation of circFBXW7 expression has been associated with increased tumor size and lymph node spread as an independent prognostic risk factor in TNBC [15]. There was no relationship between circFBXW7 levels and patient-specific characteristics and no correlation between circFBXW7 levels and tumor features. In this work, as the rare study evaluating the usage of circFBXW7 serum levels regarding BC diagnosis, we showed that circFBXW7 levels correlated with BC diagnosis (*p* = 0.043, r = 0.183). Mean levels of circFBXW7 were higher in BC patients compared with the controls (7.00 vs. 5.26), and circFBXW7 showed low expression with a Fold Change value of 0.30. We determined that circFBXW7 is a helpful biomarker in diagnosing BC with a positive predictive value of 87.6% when the cut-off value is 6.17.

A comparative study showed that circABCB10 was up-regulated in malignancies such as esophageal squamous cell carcinoma, cerebral glioma, non-small cell lung cancer, oral squamous cell carcinoma, ovarian cancer of epithelial origin, clear cell renal cell carcinoma, nasopharyngeal carcinoma, osteosarcoma, and gastric cancer. In a study on both gastric cancer cell line and tissue, it was found that circABCB10 expression levels were significantly increased compared to paracancerous and gastric mucosal epithelial cells, and circABCB10 knockdown significantly decreased cell viability and invasion ability and promoted cell apoptosis. Moreover, miR-1252-5p, whose expression is suppressed by circABCB10, negatively regulates MYC, an oncogenic gene with a well-known role in cancer. Thus, circABCB10 up-regulates MYC expression by sponging miR-1252-5p and promotes the proliferation, invasion, and clonal formation of gastric cancer cells [35]. By sponging mir-620, circABCB10 can up-regulate the expression of the FABP5 axis to promote tumoral growth and cancer cellularity in cerebral glioma and act as an oncogenic factor in glioma. It has also been observed that circABCB10 overexpression increases the proliferation of cancer cells by sponging mir-203 in esophageal cancer and increases the spread of cells by affecting the slug/E-cadherin signaling pathway, and mir-203 sponging is effective in the growth of osteosarcoma [36]. In addition, circABCB10 was found to be overexpressed in cervical cancer patients and was closely associated with lymph node metastasis, FIGO (International Federation of Gynecologists and Obstetricians) stage, and tumor size. This study revealed that circABCB10 sponges and reduces the expression of miR-128-3p, which promotes reducing cell proliferative, migratory, and invasive potential, thus preventing cancer formation and spread. That explains the high expression of circABCB10 in breast, cervix, and esophageal cancer and its relationship with lymph node metastasis, FIGO stage, and tumor size increase [37]. Furthermore, Yang W et al. revealed that circABCB10 is associated with chemoresistance in paclitaxel-resistant BC cells, which may play an oncogenic role in BC cells. This study determined that circABCB10 expression was high in paclitaxel-resistant BC cells. In the continuation of the study, it was observed that circABCB10 expression and paclitaxel resistance decreased in cancer cells as a result of intracellular transfection (suppression) of circABCB10 [38]. Liang et al. showed that circABCB10 expression increased approximately five to tenfold in cancer tissue compared to non-cancerous tissue samples. In the controlled study with increased sample size, specific siRNA targeting circABCB10 were transfected into BC cells (MCF-7 and MDA-MB-231), which led to a significant decrease in circABCB10 expression. Overall, the results confirmed the upregulation of circABCB10 in BC. circABCB10 has been shown to play an oncogene role in BC carcinogenesis through sponging miR-1271. Consequently, the knockdown of circABCB10 indicates suppressed proliferation and enhanced apoptosis in BC cells [17]. There was no relationship between circABCB10 levels and patient-specific characteristics and no correlation between circABCB10 levels and tumor features. This work showed circABCB10 levels correlated with BC diagnosis (*p* = 0.001, r = −0.291). Mean levels of circABCB10 were lower in BC patients than the controls (5.41 vs. 8.88), and circABCB10 showed high expression with a Fold Change value of 11.09. We determined that circABCB10 is a helpful biomarker in diagnosing BC with a positive predictive value of 82.4% when the cut-off value is 7.04.

Studies in the literature have shown that high expression of mir-1236 in some malignancies has tumor-suppressor properties, and especially in gastric cancer, miR-1236 is negatively regulated by circ0103552 and associated with poor prognosis in gastric cancer [39]. The study on thyroid cancer showed that circ0103552 was up-regulated in thyroid cancer tissue and cell lines. This study revealed that circ0103552 reduced the invasion and proliferation of cancer cells with its suppression by sponging miR-127 as a result. Therefore, since circ0103552 acts as a regulator through the miR-127 mechanism in thyroid cancer patients, it has also been determined to be considered a new therapeutic target in thyroid cancer treatment [40]. The study investigating the relationship between BC and circRNA showed that circ0103552 showed overexpression in BC tissue and cell lines. This study revealed that circ0103552 overexpression facilitated the proliferation, migration, and invasion of BC cells, while the knockdown of circ0103552 would have the opposite effect. The study showed that circ0103552 sponges miR-515-5p and the effect of circ0103552 is reduced or lost, which is explained by the fact that circ0103552 sponges miR-515-5p and restricts its expression in BC cells and miR-515-5p acts against the functions of circ0103552 in BC cells. In addition, CYR61 was revealed to be a downstream target of miR-515-5p in BC cells, and circ0103552 was shown to up-modulate CYR61 expression by targeting miR-515-5p. This study examined BC tissue specimens and showed that high expression of circ0103552 was associated with tumor progression characteristics such as tumor size and axilla spread [41]. A study by Liu et al. revealed that circRNA-M-TO1 inhibits BC cell proliferation and reverses monastrol resistance through the TRAF4/Eg5 pathway. Additionally, the study predicted that circ0103552 sponges several miRNAs [42]. Our study revealed circ0103552 levels correlated with BC diagnosis (*p* < 0.001, r = 0.321). According to our work, ean levels of circ0103552 were higher in BC patients than in the control group (6.41 vs. 4.45), and circ0103552 showed low expression with a Fold Change value of 0.26. circ0103552 is a helpful diagnostic agent with a positive predictive value of 88.9% when the cut-off value is 6.30. Although Huang Q et al. found that circ0103552 showed high expression in cancer tissue samples, we found that low expression was associated with tumor development in our study. Again, in studies examining circRNA levels in cancer tissue specimens, researchers have found that circFBXW7 or circ0103552 is related to aggressive BC characteristics [15,41]. We did not detect a similar relationship between tumor characteristics and the circRNAs examined. This contrast may be explained by the aim of our research to investigate whether circRNAs can be used in serum as a non-invasive diagnostic biomarker. In addition, the different results may have been due to ethnic or methodological differences.

In addition to the studies in the literature, we also performed ROC analyses to define the usefulness of circRNAs. In this study, area under curve areas were evaluated in ROC analysis and it was aimed to reveal whether these under curve values will increase or not, that is, the change of diagnostic accuracy in dual use. According to these analyses, we found that each circFBXW7, circABCB10, and circ0103552 could separately be used as an indicator in the diagnosis of BC. We also found that the diagnostic value of these circRNAs in combination becomes clearer and can reach up to 90.8%.

In the literature, imaging methods or circulating proteins such as CEA or CA15-3 have been compared with circRNAs in BC detection [43]. However, in routine practice, these circulating proteins are used for predicting cancer progression in follow-up, while other imaging methods are generally used primarily for screening. Therefore, we did not perform any additional investigation in this regard. In contrast, some studies in the literature have shown that the use of BRCA gene mutation for screening to reduce BC mortality may contribute 15–40%, but at the same time, creates a disadvantage of 5–50% overdiagnosis and overtreatment [44]. Considering that circRNAs support diagnosis at a high rate, as in our study, we can think that they can be used in this field to avoid these disadvantages.

Our study has some limitations. The first one is that the number of individuals included in the patient and control groups was not high because it was a single-center study. Secondly, the sample sizes of the patient and control groups were not similar. Another is that patient and control group characteristics may not be homogeneously distributed regarding patient-specific risk factors. The outcomes of these analyses may have produced conflicting conclusions. Finally, due to time constraints in the study process, it is inherently not possible to objectively predict that these biomarkers are helpful as prospective diagnostic tools in healthy individuals. Therefore, we need larger sample sizes and multicenter studies for validation. Since this patient group consisted of a recent patient group, it was not compared with other biomarkers. Since it was a preliminary study, no additional examination was performed for progression and treatment response. It will be evaluated in future studies. Perhaps in multicenter studies where the sample size is increased, the diagnostic role of circRNA levels in treatment follow-up and changes in treatment response can be evaluated together with other biomarkers, and the results can be generalized.

## 5. Conclusions

As far as we know, this is the first study to demonstrate a possible link between serum circFBXW7, circABCB10, and circ0103552 levels and BC. According to the results we obtained from this study, we found that BC development was affected by upregulating circABCB10 expression and downregulating circFBXW7 and circ0103552 expression in the patient group compared to the control group. In addition, our ROC analysis showed that these circRNAs can diagnose BC with high success when used separately and in combination. In conclusion, we believe that emphasizing the importance of the functions of circRNAs in BC development and drug resistance will guide future research in elucidating the molecular pathogenesis of BC and developing new treatment strategies.

## Figures and Tables

**Table 1:** circRNA levels and tumor features.

			circFBXW7		circABCB10		circ0103552	
Variables		N (%)	Mean ± STD	*p*	Mean ± STD	*p*	Mean ± STD	*p*
**Age**	<50	35 (38.0%)	5.99 ± 3.96	0.081	4.64 ± 4.52	0.254	7.76 ± 5.47	0.662
	≥50	57 (62.0%)	7.62 ± 4.48		5.88 ± 3.33		8.25 ± 4.98	
**Tumor size**	<2 cm	36 (39.1%)	6.43 ± 3.41	0.311	5.26 ± 5.10	0.828	7.50 ± 4.82	0.402
	≥2 cm	56 (60.9%)	7.37 ± 4.84		5.50 ± 5.06		8.43 ± 5.36	
**Breast cancer type**	IDC	75 (81.5%)	7.21 ± 4.38	0.335	5.54 ± 5.32	0.607	8.03 ± 5.24	0.811
	Others *	17 (18.5%)	5.08 ± 4.16		4.83 ± 3.70		8.19 ± 4.90	
**Histologic grade**	1–2	59 (64.1%)	6.99 ± 3.29	0.892	5.35 ± 4.25	0.881	7.90 ± 4.44	0.687
	3	33 (35.9%)	7.01 ± 5.83		5.50 ± 6.30		8.35 ± 6.28	
**cT**	1–2	65 (70.7%)	6.45 ± 3.78	0.058	5.06 ± 5.10	0.308	7.77 ± 4.79	0.396
	3	27 (29.3%)	8.33 ± 5.32		6.24 ± 4.93		9.77 ± 5.97	
**cN**	Negative	52 (56.5%)	6.79 ± 4.16	0.579	5.42 ± 5.09	0.872	8.04 ± 4.33	0.932
	Positive	40 (43.5%)	7.29 ± 4.61		5.39 ± 5.06		8.10 ± 6.11	
**ER**	Negative	16 (71.4%)	7.15 ± 3.54	0.890	6.05 ± 4.45	0.591	6.11 ± 5.86	0.109
	Positive	76 (82.6%)	6.97 ± 4.50		5.28 ± 5.17		8.44 ± 4.95	
**PR**	Negative	20 (21.7%)	6.72 ± 3.29	0.742	5.47 ± 4.13	0.954	6.57 ± 5.80	0.144
	Positive	72 (78.3%)	7.08 ± 4.61		5.39 ± 5.30		8.48 ± 4.92	
**HER2**	Negative	72 (78.3%)	7.04 ± 4.48	0.869	5.36 ± 5.16	0.867	8.17 ± 4.93	0.704
	Positive	20 (21.7%)	6.86 ± 3.92		5.57 ± 4.75		7.67 ± 5.98	
**Receptor status**	Luminal	76 (82.6%)	7.01 ± 4.52	0.941	5.32 ± 5.20	0.715	8.42 ± 4.98	0.144
	Non-luminal	16 (17.4%)	6.97 ± 3.49		5.83 ± 4.39		6.35 ± 5.74	
**Tumor stage**	1–2	79 (85.9)	6.71 ± 3.89	0.108	5.14 ± 4.90	0.211	7.73 ± 4.77	0.129
	3	13 (14.1%)	8.80 ± 6.38		7.04 ± 5.83		10.08 ± 6.92	

Student’s T-test, Mann–Whitney U test, * Other breast cancer types include fourteen invasive lobular carcinoma, two invasive mucinous carcinoma, and one invasive tubular carcinoma.

**Table 2:** Serum assay levels in patients with breast cancer and healthy controls.

circRNA	ΔCt		Standard Deviation		2^−ΔΔCt^		Fold Change	*p*
	Patients	Controls	Patients	Controls	Patients	Controls		
circFBXW7	7.00	5.26	4.34	3.19	0.0078	0.0260	0.30	0.0102
circABCB10	5.41	8.88	5.05	4.80	0.0235	0.0021	11.09	0.0009
circ0103552	6.41	4.45	8.66	4.80	0.0117	0.0457	0.26	0.0002

Student’s T-test, Mann–Whitney U test.

**Table 3:** Outcomes of the ROC analysis of circFBXW7, circABCB10, and circ0103552.

Assay	Cut-Off Value	Sensitivity	Specificity	PPV	NPV	AUC (CI)
*circFBXW7*	6.17	71.0%	30.6%	87.6%	44.0%	0.655 (0.548–0.761)
*circABCB10*	7.70	67.4%	51.3%	82.4%	34.5%	0.700 (0.588–0.812)
*circ0103552*	6.30	87.1%	67.7%	88.9%	54.7%	0.767 (0.685–0.850)

AUC: area under the curve; CI: confidence interval; PPV: positive predictive value; NPV: negative predictive value.

**Table 4:** ROC analysis outcomes for the combined use of markers.

			Asymptotic 95% Confidence Interval	
Test Variables	AUC	Asymptotic Sig	Lower Bound	Upper Bound
*circABCB10*—*circFBXW7*	0.896	<0.001	0.796	0.947
*circ0103552*—*circABCB10*	0.908	<0.001	0.803	0.978
*circFBXW7*—*circ0103552*	0.884	<0.001	0.788	0.935

AUC: area under the curve.

## Data Availability

Not applicable.
